# A New Real-Time PCR for the Detection of *Plasmodium ovale wallikeri*

**DOI:** 10.1371/journal.pone.0048033

**Published:** 2012-10-24

**Authors:** Adriana Calderaro, Giovanna Piccolo, Chiara Gorrini, Sara Montecchini, Sabina Rossi, Maria Cristina Medici, Carlo Chezzi, Georges Snounou

**Affiliations:** 1 Department of Pathology and Laboratory Medicine, Section of Microbiology, University of Parma, Parma, Italy; 2 Université Pierre et Marie Curie - Paris VI, UMR S 945, Paris, France; 3 Institut National de la Santé et de la Recherche Médicale UMR S 945, Paris, France; Walter & Eliza Hall Institute, Australia

## Abstract

It has been proposed that ovale malaria in humans is caused by two closely related but distinct species of malaria parasites: *P*. *ovale curtisi* and *P*. *ovale wallikeri*. We have extended and optimized a Real-time PCR assay targeting the parasite’s small subunit ribosomal RNA (ssrRNA) gene to detect both these species. When the assay was applied to 31 archival blood samples from patients diagnosed with *P. ovale*, it was found that the infection in 20 was due to *P*. *ovale curtisi* and in the remaining 11 to *P*. *ovale wallikeri*. Thus, this assay provides a useful tool that can be applied to epidemiological investigations of the two newly recognized distinct *P*. *ovale* species, that might reveal if these species also differ in their clinical manifestation, drugs susceptibility and relapse periodicity. The results presented confirm that *P. ovale wallikeri* is not confined to Southeast Asia, since the majority of the patients analyzed in this study had acquired their *P*. *ovale* infection in African countries, mostly situated in West Africa.

## Introduction

*Plasmodium ovale*, most probably first observed in 1900 by Charles F. Craig [1], was formally described and named by Stephens in 1922 because of characteristic oval-shaped infected erythrocytes seen in the blood of soldier on his return to England from East African [2]. Morphologically, *P. ovale* differs only slightly from *P. vivax*, making it difficult to distinguish these two species by microscopic examination. This delayed wide acceptance by the malaria community that *P. ovale* was indeed a distinct species and not a strain of *P. vivax*. Detailed morphological and biological observations of the course of *P. ovale* infection in experimentally infected neurosyphilitic patients and in mosquitoes brought conclusive evidence that it was indeed a species in its own right [3]. The biology, pathology and epidemiology of *P. ovale* were recently reviewed [4], [5]. Briefly, the parasite causes a clinically benign infection where peak parasitaemia does not generally exceed 10,000 parasites per µl, the infection often resolves spontaneously and the relapses due to the activation of hypnozoites are clinically mild and of lesser frequency that those in *P. vivax* infections. Long considered a parasite confined to Africa it has been recorded in all endemic areas, except America [6]. Nonetheless, its prevalence in sub-Saharan Africa, even in western Africa, rarely exceeds 5%, and it is only occasionally recorded in most of the endemic areas outside Africa.

The advent of molecular assays based on the amplification of genes specific to each parasite, most often the small subunit ribosomal RNA (ssrRNA), has greatly improved and the sensitivity of detection and the identification accuracy of *P*. *ovale* [7], [8]. The first epidemiological studies revealed that figures for the prevalence of *P. ovale* derived from microscopic examination of blood smears were likely to be a gross underestimate of the true values [8], [9], [10]. The observation that some isolates microscopically identified as *P*. *ovale* could not be detected by some of the assays based on the amplification of the ssrRNA gene (reviewed in [11]) led to the discovery that this gene in *P. ovale* occurred as one or other of two types, termed classic and variant [11], [12], [13]. The occurrence of two sequence types was also found for the other *P. ovale* genes that were analyzed [13], [14], [15]. Recently, two groups working independently (United Kingdom and Thailand) added to this data and concluded that these two variant types defined two distinct non-recombining sympatric species, a proposal made in a joint publication [16], in which they were named the classic type *P. ovale curtisi* and the variant type *P. ovale wallikeri*.

The existence of such two species raises a number of important epidemiological and biological questions that are best answered by gathering data from large numbers of cases infected by these species and from mosquitoes that transmit them. This requires a molecular assay for their detection and accurate identification. In a previous study [11], we had designed novel oligonucleotide primer combinations for use in a nested-PCR assay (NPCR) that could indiscriminately detect both *P*. *ovale curtisi* and *P*. *ovale wallikeri*. Moreover, in the same study it was observed that a real-time PCR assay (Rt-PCR) previously described by us [17] and currently used in our laboratory for the diagnosis of malaria, was able to detect *P*. *ovale curtisi* but not *P. ovale wallikeri*.

An Rt-PCR assay, recently described to distinguish between the two *P. ovale* species [18], is based on distinguishing the 120 bp fragments from the reticulocyte binding protein 2 (*rbp2*) gene from the two species by a difference in their melting temperature due to six species-specific single nucleotide polymorphisms. The sensitivity of that assay was not determined, though the results were concordant with a nested PCR based on another parasite gene, the *P. ovale* sp. tryptophan-rich antigen [18]. We wished to develop an Rt-PCR assay for *P. ovale wallikeri* based on the same highly conserved non-protein coding ssrRNA gene, that is the target of our previously developed Rt-PCR assays that we routinely use for the detection of the other *Plasmodium* species [11], [17].

## Materials and Methods

### Samples

A set of 31 blood samples, collected on hospital admission from patients presenting with symptoms consistent with malaria between January 1999 and December 2011, was used. These samples were selected from a larger set of samples because they had been found to contain *P. ovale* from previous studies [11], [17], [19]. Parasitaemia was calculated as previously described [17], [20] from thin smears, when these were available. Genomic DNA was prepared from a 200 µl of each sample using the “High Pure Template Preparation kit” (Roche, Indianapolis, USA) according to the manufacturer’s instructions [17].

### Ethical Approval

The samples analyzed in this and previous studies had been obtained by the University Hospital of Parma for routine diagnosis purposes, as such no approval by the local review committee was required because of the laboratory diagnosis results had been reported in the medical records of the patients as a diagnostic answer to a clinical suspicion of malaria, and ethical approval at the University Hospital of Parma is required only in cases where the clinical samples are to be used for applications other than diagnosis. According to Italian Ministry of Health guidelines on malaria laboratory diagnosis, only verbal informed consent is requested of the patients on hospital admission. At the University Hospital of Parma informed consent procedures for the laboratory diagnosis of infectious diseases other than HIV serology do not require local committee approval because they are included in the Italian Public Health Legislation. In order to document the process, physicians must write a medical order with the personal and clinical data of each patient who asks for health assistance in a public hospital including the University Hospital of Parma. Blood samples were analyzed anonymously both for microscopy and PCR assays.

### Amplification Assays

The ssrRNA genes were the targets of all amplifications. All the 31 purified DNAs were submitted to Po-Pov NPCR and to a Real-time PCR specific for *P*. *ovale curtisi* (Po Rt-PCR) as reported previously [11], [17]. The identification of species other than *P*. *ovale* was performed by nested PCR (NP-2002) as previously described [21]. For all these assays 5 µl of template was used to initiate the amplification reactions.

The new primer OVA-Fv, 5′-TTTTGAAGAATATATTAGGATACATTATAG-3′ employed in association with the previously described primer OVA-R [17], and the new probe Ovav, 5′-FAM-CCTTTTCCCTTTTCTACTTAATTCGCTATTCATG-TAMRA-3, were used to identify *P*. *ovale wallikeri* by Real-time PCR assay (Pov Rt-PCR). The new primer and probe were designed based on the *P*. *ovale* ssrRNA gene sequence Accession Number AJ001527.

Purified DNAs were amplified in an ABI PRISM 7300 Real-time PCR system (SDS version 1.3.1) (Applied Biosystems, Foster City, CA, USA) instrument. Briefly, the reaction was performed in a 50 µl PCR mixture containing 5 µl of template, 25 µl of TaqMan 2X (Applied Biosystems) universal PCR master mix, a 600 nM concentration of each parasite species-specific primer set, and a 250 nM concentration of each corresponding probe. Amplification and detection were performed under the following conditions: 2 min at 50°C to achieve optimal AmpErase uracil-N-glycosylase activity, 10 min at 95°C to activate the AmpliTaq Gold DNA polymerase, and 55 cycles of 15 s at 95°C and 1 min at 60°C. Each experiment included at least one reaction mixture without DNA as a negative control, and each specimen was run in duplicate.

The specificity of the new Pov Rt-PCR was tested by using purified DNA obtained by blood samples from 10 healthy individuals and by 9 blood samples PCR-confirmed to be positive for *P*. *falciparum*, 9 for *P*. *vivax* and 5 for *P*. *malariae* and a synthetic DNA oligonucleotide containing a target sequence of *P. knowlesi* ssrRNA gene (synthesized by TIB Molbiol S.r.l., Genova, Italy). Moreover, genomic DNAs from *in vitro* culture samples from other blood protozoa such as *Toxoplasma gondii* and *Leishmania infantum* were also used.

## Results and Discussion

The 31 samples included in this study (Table 1) formed a subset of samples collected prior to anti-malarial treatment from patients presenting to the University Hospital of Parma. Although initial routine microscopy had failed to detect *P. ovale* in many, the presence of this parasite was established by NPCR in previous studies [11], [17]. The parasitaemias ranged from <50 to 20,500 parasites/microlitre. All 31 samples were *P*. *ovale* positive by the NPCR assay specifically developed to detect either of the two *P. ovale* species [11]. The samples were negative for *Plasmodium* species other than *P. ovale*, except No. 11 and 14 which were also positive for *P*. *falciparum* and *P. malariae*, and No. 22 which was also positive for *P. falciparum*, as established by nested PCR assay [21].

**Table 1:** Characteristics of *P*. *ovale* samples and species identification by microscopy, nested PCR and Real time PCR.

No.	Country visited	Latency^a^ (mo)	Parasites/µl	Microscopy^b^	Nested PCR^c^	Po^c^ Rt-PCR & Pov Rt-PCR
1	Senegal	5	–	N	Po	*P. o. curtisi*
2	Ghana	NA	<500	Pf	Po	*P. o. curtisi*
3	Africa	24	3,200	Pv	Po	*P. o. curtisi*
4	Mozambique	NA	10,000	Pv	Po	*P. o. curtisi*
5	unknown	NA	5,000	Pv	Po	*P. o. curtisi*
6	unknown	NA	<5,000	Pv	Po	*P. o. curtisi*
7	Ghana	NA	7,000	Pv	Po	*P. o. curtisi*
8	Cameroon	5	<500	Po	Po	*P. o. curtisi*
9	Cameroon	3	6,000	Po	Po	*P. o. curtisi*
10	Nigeria	0.1	12,000	Po	Po	*P. o. curtisi*
11	Burkina Faso	0.25	<500	*Plas.* sp.	Po+Pf+Pm	*P. o. curtisi*
12	Ivory Coast	NA	<5,000	Pv	Po	*P. o. curtisi*
13	unknown	NA	<200	Pv or Po	Po	*P. o. wallikeri*
14	Burkina Faso	NA	<50	Pf	Po+Pf+Pm	*P. o. curtisi*
15	Tanzania	NA	<5,000	Po	Po	*P. o. wallikeri*
16	Ghana	NA	15,000	Pv	Po	*P. o. wallikeri*
17	unknown	NA	10,000	Po	Po	*P. o. wallikeri*
18	Cameroon	6	3,500	Po	Po	*P. o. wallikeri*
19	Cameroon	NA	10,000	Po	Po	*P. o. wallikeri*
20	Ivory Coast	NA	1,850	Po	Po	*P. o. wallikeri*
21	Nigeria	12	20,500	Po	Po	*P. o. curtisi*
22	Ivory Coast	0	6,000	*Plas.* sp.	Po+Pf	*P. o. wallikeri*
23	Cameroon	9	500	*Plas.* sp.	Po	*P. o. wallikeri*
24	Ivory Coast	4.5	<500	Po	Po	*P. o. curtisi*
25	Senegal	NA	20,000	Po	Po	*P. o. wallikeri*
26	Nigeria	0.5	<50	*Plas.* sp.	Po	*P. o. curtisi*
27	Guinea	NA	<50	*Plas.* sp.	Po	*P. o. curtisi*
28	Nigeria	NA	7,000	Po	Po	*P. o. curtisi*
29	Nigeria	0.5	4,150	Po	Po	*P. o. curtisi*
30	Congo	1.4	<500	*Plas.* sp.	Po	*P. o. wallikeri*
31	Burkina Faso	10	14,500	Po	Po	*P. o. curtisi*

a The period in months (mo) between the last date in the country endemic for malaria and presentation to the hospital in Italy; NA  =  not available.

b *Plas*. sp.  =  *Plasmodium* species; Pf  =  *P. falciparum*; Pv  =  *P. vivax*; Pm  =  *P. malariae*; Po  =  *P. ovale*; N  =  negative.

c The presence of the species *P. falciparum*, *P. vivax* and *P. malariae* was determined by the classical nested PCR (NP-2002) as previously described [21], and that of *P. ovale* (*P*. *ovale curtisi* and/or *P*. *ovale wallikeri*) was determined by nested PCR using recently described oligonucleotides that amplify both of the two species [11].

Following optimization of the reaction conditions, the Rt-PCR assay for *P. ovale curtisi* and the new one for *P. ovale wallikeri* were evaluated at least in duplicate for their analytical sensitivity using serial dilution of purified plasmids into which the respective ssrRNA gene amplicons were cloned. Using a ten-fold dilution series with plasmid concentrations ranging 5×10^8^ copies per assay to a theoretical value of 0.01 copies, only template aliquots with 50 or more target copies were consistently positive (Ct values ranging from 16.7 to 38 for the highest concentration to the lowest). We noted that for assays with template aliquots containing 5 copies (whose presence was confirmed by NPCR) Ct values between 43 and 54 were obtained in >50% of the plasmid DNA replicates tested. This led us to conduct the assay over 55 cycles. We are aware that Ct values above 40 are generally considered of doubtful significance. However, according to the results we obtained by testing both clinical samples and 5 copies plasmid DNA a cut-off could be established at 54 cycles. Moreover, we feel confident of the specificity of the assay and that false positive results would be rare, since no signal could be detected by either the two *P*. *ovale*-specific primer/probe combinations when test assays were initiated with DNA obtained from healthy individuals or from individuals infected with *P. falciparum* (n = 9), *P. vivax* (n = 9), *P. malariae* (n = 5), the non-corresponding *P. ovale* species, or with a *P. knowlesi* DNA oligonucleotide corresponding to the target (data not shown). Moreover, no cross-reactivity could be detected with assay using genomic DNA purified from *Toxoplasma gondii* or *Leishmania infantum*, neither was a signal detected for any of the no template assays (data not shown).

The different PCR assays were applied to the genomic DNAs purified from the 31 samples collected prior to anti-malarial treatment from 31 patients presenting to the University Hospital of Parma. Twenty of these samples were found infected with *P. ovale curtisi* and 11 with the *P*. *ovale wallikeri* (Table 1). On a single occasion Ct values 53.47 and 50.57 were obtained in duplicate *P*. *ovale wallikeri* runs using template isolated from a sample, No. 30, with a very low parasitaemia (Table 1). In this case, the presence of *P. ovale wallikeri* was confirmed by nested PCR assay. A similar result was observed with sample No. 11 (*P*. *ovale curtisi*) (Table 1) in which the nested-PCR assay also confirmed the triple infection with *P*. *falciparum* and *P*. *malariae*.

The results we present concord with the initial observations on the two *P. ovale* species [16], [18], namely that the *P*. *ovale wallikeri* (previously known as the variant type) is not confined to southeast Asia since nearly all the patients acquired the infection in Africa, and that the two species are generally sympatric in the countries where they occur. Mixed infections with both *P. ovale* species were not found in any of the samples we have analyzed. Finally, it should be pointed out that nine of the patients presented to the hospital between three months and two years after their arrival to Italy. Thus, it is likely that the samples from these patients contained parasites from relapses, i.e. blood stage infections originating from the activation of a subset of the hypnozoites in the liver. This would indicate that relapses seem to occur in both species, though the low number of patients for which the period of latency was available precluded statistically significant comparisons of the relapse patterns of the two species.

The ability to detect and distinguish the two *P*. *ovale* species using the Rt-PCR protocol presented here opens the way to epidemiological investigations of these parasites. As a matter of fact, *P*. *ovale* is one of the least studied of the *Plasmodium* species that infect humans. In the context of the goal of malaria elimination and eventual eradication, it becomes important to investigate *P*. *ovale*, given that its prevalence is likely to be substantially higher than previously thought and that it can maintain itself in the human host for long periods as a result of its capacity to produce hypnozoites. Any meaningful investigations of the true epidemiology and biology of the two *P*. *ovale* species, whose infections lead to only relatively scanty parasitaemias (peaks of less than 10,000 parasites/µl) even in primary infections [4], will necessitate the application of sensitive and specific molecular methods of detection.
